# Prognostic impact of proton pump inhibitors for immunotherapy in advanced urothelial carcinoma

**DOI:** 10.1002/bco2.118

**Published:** 2021-10-08

**Authors:** Yoshiharu Okuyama, Shingo Hatakeyama, Kazuyuki Numakura, Takuma Narita, Toshikazu Tanaka, Yuki Miura, Daichi Sasaki, Daisuke Noro, Noriko Tokui, Teppei Okamoto, Hayato Yamamoto, Shintaro Narita, Takahiro Yoneyama, Yasuhiro Hashimoto, Tomonori Habuchi, Chikara Ohyama

**Affiliations:** ^1^ Department of Urology Hirosaki University Graduate School of Medicine Hirosaki Japan; ^2^ Department of Urology Akita University Graduate School of Medicine Akita Japan; ^3^ Department of Urology Aomori Prefectural Central Hospital Aomori Japan; ^4^ Department of Urology Mutsu General Hospital Mutsu Japan; ^5^ Department of Urology Odate Municipal Hospital Odate Japan; ^6^ Department of Advanced Transplant and Regenerative Medicine Hirosaki University Graduate School of Medicine Hirosaki Japan

**Keywords:** antibiotics, immune checkpoint inhibitors, immunotherapy, prognosis, proton pump inhibitor, urothelial carcinoma

## Abstract

**Objective:**

To evaluate the effects of the concomitant use of proton pump inhibitors (PPIs) and/or antibiotics (Abs) on oncological outcomes in patients with advanced urothelial carcinoma.

**Patients and methods:**

We retrospectively evaluated 155 patients with advanced urothelial carcinoma who were treated with immune checkpoint inhibitors (ICIs) between August 2015 and April 2021. The concomitant use of PPI or Abs was defined as any PPI or Abs administered within 30 days before ICI initiation and during ICI therapy. The primary outcomes were the effect of PPI and/or Abs use on the objective response rate (ORR) and immune‐related adverse events (irAEs). The secondary outcomes were the effects of PPI and/or Abs use on progression‐free survival (PFS) and overall survival (OS) after ICI therapy analyzed using the inverse probability of treatment weighting‐adjusted Cox regression analysis.

**Results:**

Of the 155 patients enrolled in the study, 99 (64%) were PPI users and 56 (36%) Abs users. PPI users were associated with a significantly poorer ORR than non‐PPI users (41% vs. 20%, respectively), whereas Abs use was not significantly associated with changes in ORR. The rate of irAEs was not significantly associated with the use of PPIs or Abs. Multivariate inverse probability of treatment weighting‐adjusted Cox regression analysis revealed significantly poorer PFS and OS in PPI users than in non‐PPI users, whereas Abs use was not associated with poorer outcomes.

**Conclusion:**

The concomitant use of PPI may adversely affect oncological outcomes in patients with locally advanced or metastatic urothelial carcinoma treated with ICI therapy.

## 1 INTRODUCTION

Locally advanced or metastatic urothelial carcinoma (UC) is a life‐threatening disease with a poor prognosis.^1^, ^2^, ^3^ Although immune checkpoint inhibitors (ICIs) have significantly impacted treatment strategies in UC in both clinical trials and real‐world practice,^4^, ^5^, ^6^, ^7^, ^8^ the heterogeneity of treatment responses and outcomes is a major problem.^9^, ^10^, ^11^ Proton pump inhibitors (PPIs) are common chronic medications used for gastroesophageal reflux and/or peptic ulcers. However, the use of PPI may cause gut microbiota dysbiosis, driven by both altered stomach acidity and direct compound effects.^12^ A recent study indicated that PPI use can cause significantly poor outcomes in patients with advanced UC treated with ICI therapy, based on pooled ad‐hoc analysis of data from the IMvigor210 (single‐arm atezolizumab trial, *n* = 429) and IMvigor211 (Phase III randomized trial of atezolizumab vs. chemotherapy, *n* = 931) clinical trials.^13^ Also, the same pooled ad‐hoc analysis of data from IMvigor210 and IMvigor211 showed that concomitant use of antibiotics (Abs) can also reduce the effectiveness of cancer immunotherapies.^14^ However, several studies have reported conflicting results regarding the influence of PPI on the efficacy of ICIs in advanced non–small‐cell lung cancer and melanoma.^15^, ^16^, ^17^, ^18^ Furthermore, no previous study has investigated the association of PPI and/or Abs with prognosis in patients with advanced UC treated with ICI therapy. Therefore, we investigated the effect of PPI and/or Abs use on oncological outcomes in patients with advanced UC.

## 2 PATIENTS AND METHODS

### 2.1 Design and ethics statement

This retrospective, multicenter study was performed in accordance with the principles of the Declaration of Helsinki and was approved by the ethics committee of the Hirosaki University School of Medicine (2019–099) and all hospitals included in this study. Written consent was not obtained in exchange for public disclosure of study information (opt‐out approach).

### 2.2 Patient selection and demographics

We retrospectively evaluated consecutive 160 patients with locally advanced (cT4 or pN+) or metastatic UC treated with ICI therapy between August 2015 and April 2021 at two academic centers and three general hospitals. We verified our retrospective data before analysis. Five patients who were treated within 3 months or who had no imaging evaluation were excluded. Thus, 155 patients with advanced UC were included. The concomitant use of PPI or Abs was defined as any PPI or Abs administered within 30 days before ICI initiation and during ICI therapy. A single dose of first‐generation cephem antibiotics (e.g., cephalexin via intravenous injection) were administrated to the majority of patients for peri‐procedural purposes (e.g., biopsy or transurethral resection of the bladder tumor) as a standard of care. The following variables were recorded and evaluated: age, sex, Eastern Cooperative Oncology Group performance status (ECOG‐PS), estimated glomerular filtration rate (eGFR), clinical stage, progression‐free survival (PFS), and overall survival (OS). Tumor stage and grade were stratified based on the eight editions of the TNM classification.^19^ The indication of PPI was the treatment of gastroesophageal reflux and/or peptic ulcers.

### 2.3 Platinum‐based first‐line chemotherapy

Patients received either gemcitabine plus cisplatin; gemcitabine plus carboplatin; or methotrexate, vinblastine, doxorubicin, and cisplatin.^2^, ^20^ Cycles were repeated every 21 or 28 days until disease progression or intolerable adverse events. Because of the population difference, we used the modified cisplatin ineligibility criteria presented by Galsky et al.^21^ Using the original criteria, a patient was defined as cisplatin ineligible if at least one of the following criteria was met: ECOG‐PS > 1, eGFR <60 ml/min/1.73 m^2^, grade >1 hearing loss, grade >1 neuropathy, and/or New York Heart Association (NYHA) Class III heart failure. In addition, we defined the marginal criteria as being ECOG‐PS 1, eGFR 50–60 ml/min/1.73 m^2^, NYHA Class II heart failure, and age >75–80 years. Patients with two or more marginal factors (such as age ECOG‐PS 1 and eGFR 55 ml/min/1.73 m^2^) were classified as cisplatin ineligible.

### 2.4 Outcomes

We divided the patients into two groups based on the concomitant use of PPI or Abs. The primary outcomes were the effects of PPI and/or Abs use on the objective response rates (ORRs) and immune‐related adverse events (irAEs). The secondary outcomes were the effects of PPI and/or Abs use on PFS and OS after ICI therapy.

### 2.5 Statistical analyses

We performed statistical analyses using BellCurve for Excel version 3.10 (Social Survey Research Information Co., Ltd., Tokyo, Japan), GraphPad Prism version 7.00 (GraphPad Software, San Diego, CA, USA), and R: 4.0.2, A Language and Environment for Statistical Computing (The R Foundation, Vienna, Austria). Intergroup differences were examined using Student's *t* test or the Mann–Whitney *U* test. Fisher's exact test or the *χ* ^2^ test was used to compare categorical variables. Quantitative variables were expressed as means with standard deviations or medians with interquartile ranges. The rate of OS from ICI therapy until death was estimated using the log‐rank test. The effects of PPI use on PFS and OS after ICI therapy were determined using multivariable Cox regression analyses via the inverse probability of treatment weighting (IPTW) method. The hazard ratio (HR) with a 95% confidence interval (95% CI) was calculated after controlling for potential confounders, including patient age, sex, ICI therapy treatment line, ECOG‐PS at initiation of ICI therapy, tumor type, PPI use, Abs use, and exposure to radiotherapy.

## 3 RESULTS

### 3.1 Baseline characteristics

Table 1 presents the baseline characteristics of the patients. The median patient age was 72 (64–79) years. Of the 155 patients, 145 (94%) were treated with pembrolizumab. Overall, 56 and 99 patients were non‐PPI users and PPI users, respectively. The majority of patients (*n* = 79, 80%) used PPIs before ICI therapy, suggesting a long‐term continuous medication. There were no significant differences in baseline characteristics between the non‐PPI users and PPI users. The proportions of patients with concomitant use of Abs were 38% and 51% in non‐PPI users and PPI users, respectively. Of the PPI users (*n* = 99), 79 (80%) patients had been using PPI continuously for 3 months before the initiation of ICI therapy.

**TABLE 1:** Baseline characteristics at initiation of immune checkpoint inhibitors (ICI) therapy

	Non‐PPI users	PPI users	*p* value
*n*	56	99	
Age, years (IQR)	73 (65–81)	71 (63–78)	0.158
Male, *n*	43 (77%)	75 (76%)	0.886
UTUC, *n*	22 (39%)	43 (43%)	0.617
ECOG PS > 1	11 (20%)	23 (23%)	0.689
Antibiotics (Abs) users	21 (38%)	50 (51%)	0.119
PPI use before the ICI therapy, *n*	0 (0%)	79 (80%)	
Local therapy, *n*	37 (66%)	54 (55%)	0.178
Surgery	28 (50%)	45 (45%)	0.590
Radiotherapy	13 (23%)	13 (13%)	0.132
Outcomes of first‐line therapy			
Carboplatin‐based regimens, *n*	34 (61%)	65 (66%)	0.743
Number of cycles (IQR)	2 (2–4)	3 (2–3)	0.329
Objective response, *n*	16 (29%)	30 (30%)	0.839
Treatment line of ICI therapy (IQR)	2 (2–2)	2 (2–2)	0.185
Type of ICI therapy (PD‐1 vs. PD‐L1)			0.620
Pembrolizumab	49 (88%)	96 (97%)	
Nivolumab	5 (8.9%)	1 (1%)	
Atezolizumab	1 (1.8%)	1 (1%)	
Durvalumab	1 (1.8%)	1 (1%)	
Clinical TNM stage, *n*			
T4	15 (27%)	38 (38%)	0.494
N+	33 (59%)	64 (65%)	0.439
M1	38 (68%)	67 (68%)	0.982
Number of metastatic sites	1 (1–2)	1 (1–2)	0.355
Number of metastatic sites >1	18 (32%)	38 (31%)	0.436
eGFR (ml/min/1.73 m^2^) (IQR)	48 (40–70)	51 (40–64)	0.760
Concomitant use of antibiotics (Abs), *n*	21 (38%)	50 (51%)	0.118
Disease progression after ICI therapy, *n*	26 (46%)	70 (71%)	
Deceased, *n*	21 (38%)	60 (61%)	

eGFR, estimated glomerular filtration rate; ICI, immune checkpoint inhibitor; IQR, interquartile range; PD‐1, programmed cell death 1; PD‐L1, programmed cell death ligand 1; PPI, proton pump inhibitor; UTUC, upper tract urothelial carcinoma.

### 3.2 Primary outcomes

**FIGURE 1:**
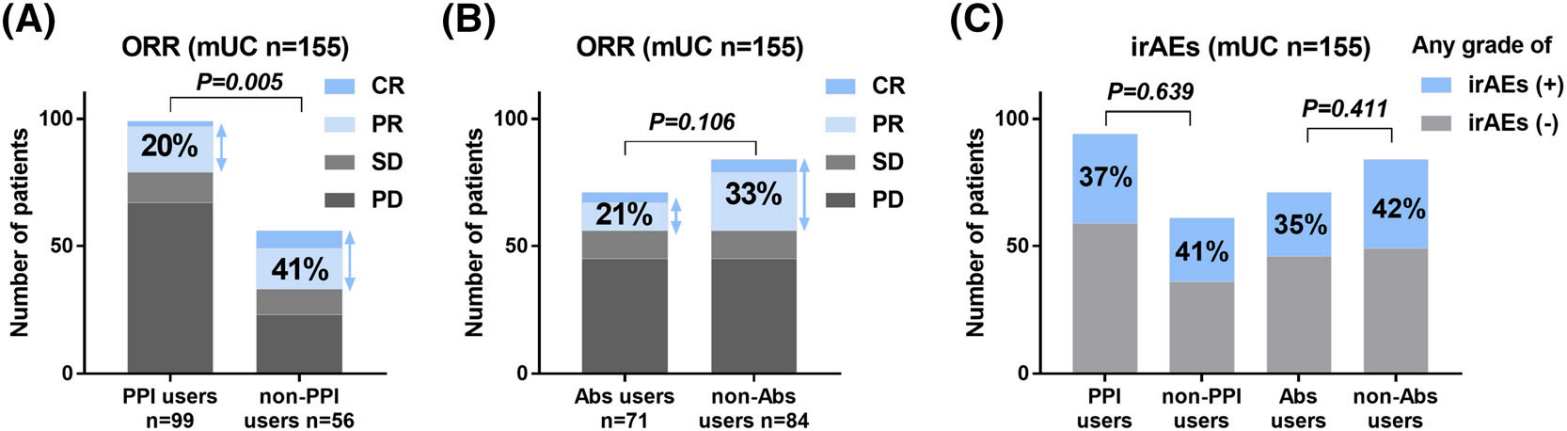
Object response rate (ORR) and occurrence of immune‐related adverse events (irAEs). ORR were compared between the proton pump inhibitor (PPI) users and non‐PPI users (A). ORRs were compared between the antibiotic (Abs) users and non‐Abs users (B). Occurrences of irAEs were compared between the PPI users and non‐PPI users, and between the Abs users and non‐Abs users (C)

The ORR was significantly increased in non‐PPI users (41%) compared that in PPI users (20%) (Figure 1A, *p* = 0.005). ORR was not significantly different between the Abs users (21%) and non‐Abs users (33%) (Figure 1B, *p* = 0.106). The rate of irAEs was not significantly different between the PPI users and non‐PPI users (*p* = 0.639) (Figure 1C).

### 3.3 Secondary outcomes

#### 3.3.1 Effects of the concomitant use of PPI or Abs on oncological outcomes

**FIGURE 2:**
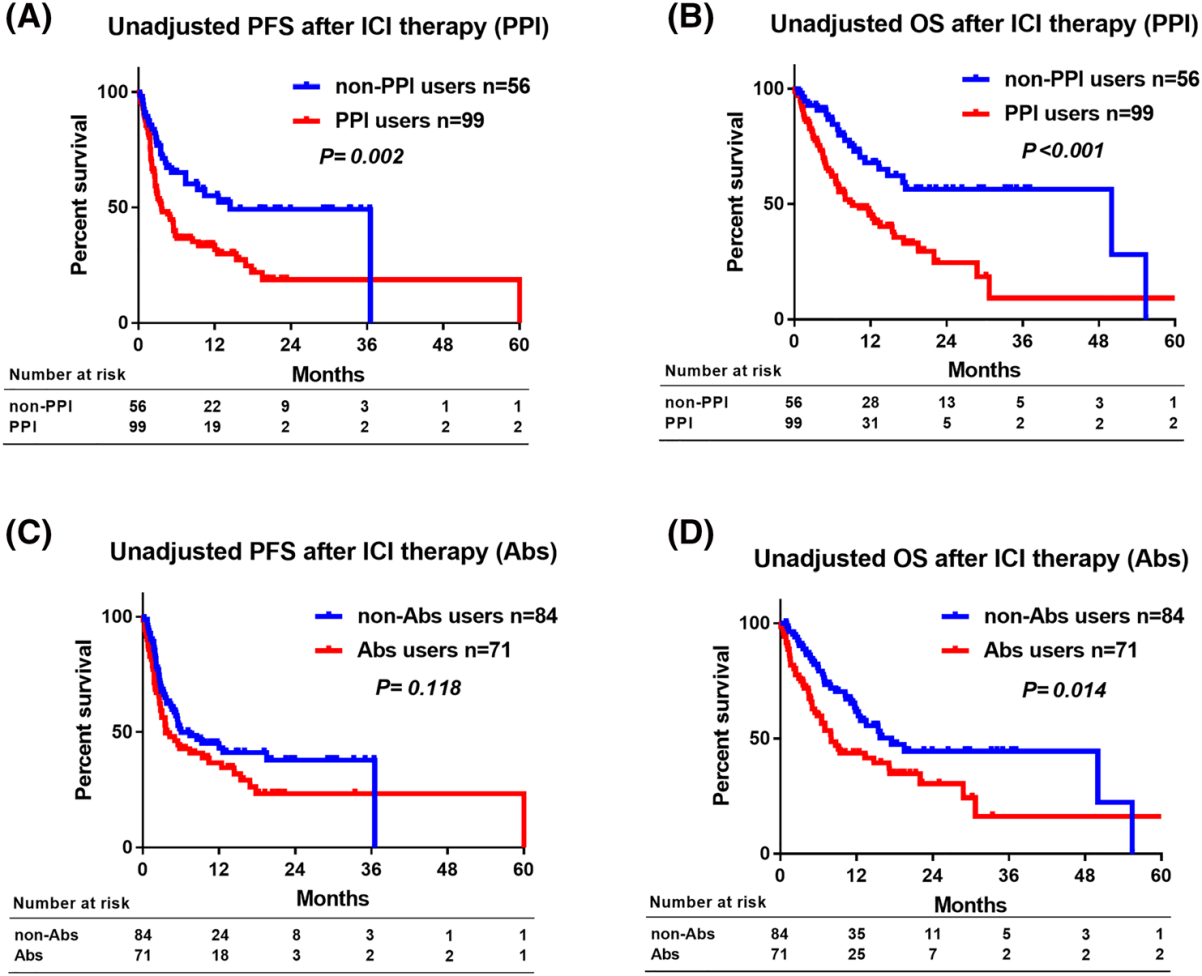
The prognostic impact of proton pump inhibitor (PPI) or antibiotic (Abs) in patients treated with immune checkpoint inhibitor (ICI) therapy. An unadjusted comparison of progression‐free survival (PFS) (A) and overall survival (OS) (B) after ICI therapy between PPI users and non‐PPI users. An unadjusted comparison of PFS (C) and OS (D) after ICI therapy between Abs users and non‐Abs users

The PFS after ICI therapy was significantly longer in non‐PPI users than in PPI users (median 14 vs. 3.6 months, *p* = 0.002) (Figure 2A). The OS after ICI therapy was significantly longer in non‐PPI users than in PPI users (median 50 vs. 9.1 months, *p* < 0.001) (Figure 2B). The PFS after ICI therapy was not significantly different between the non‐Abs and Abs users (median 6 vs. 3.6 months, *p* = 0.118) (Figure 2C). The OS after ICI therapy was significantly different between the non‐Abs and Abs users (median 18 vs. 8 months, *p* = 0.014) (Figure 2D).

#### 3.3.2 The effect of concomitant use of PPI and Abs on oncological outcomes

**FIGURE 3:**
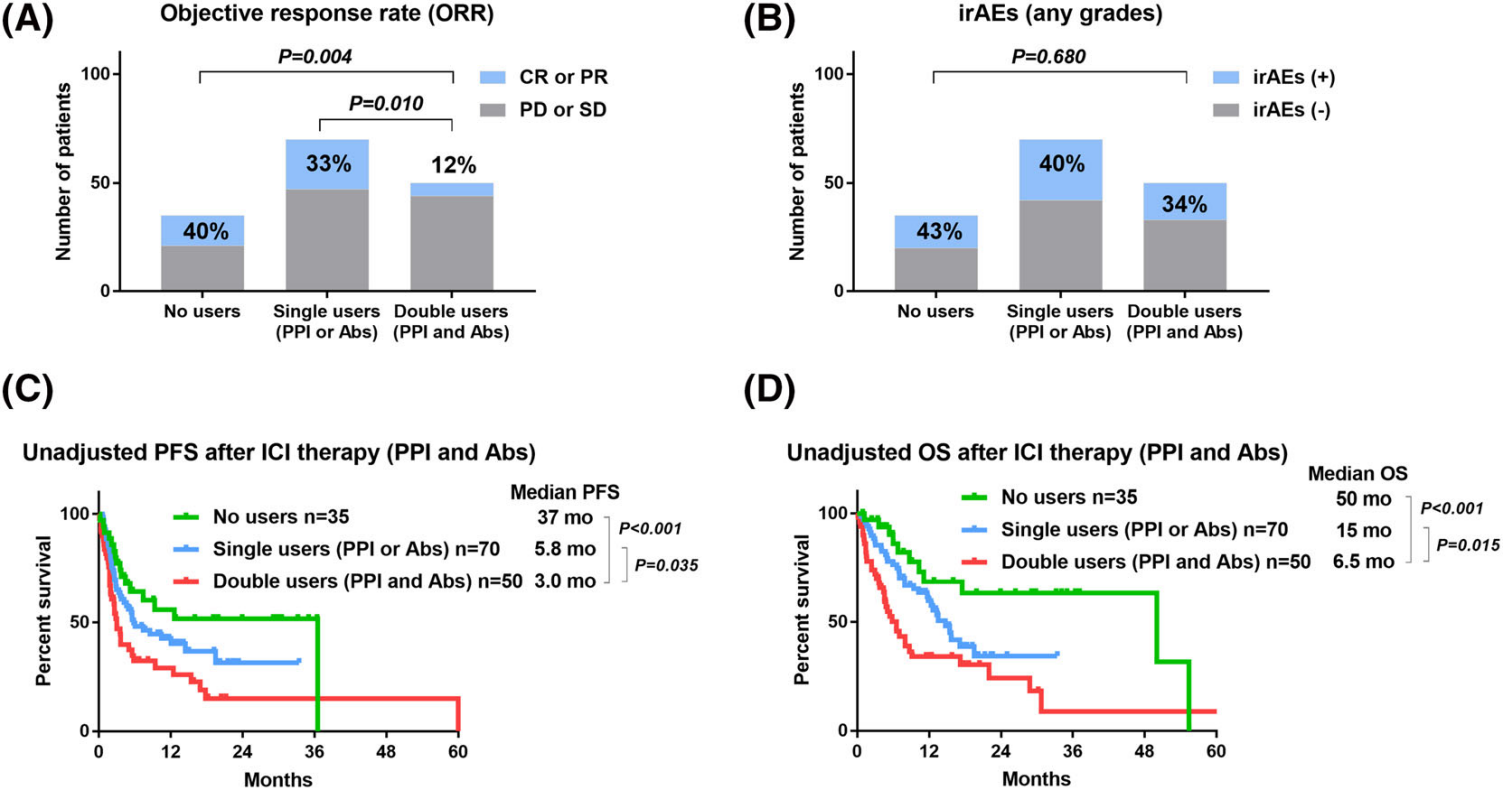
The impact of concomitant users of proton pump inhibitor (PPI) and antibiotic (Abs) on object response rates (ORRs), immune‐related adverse events (irAEs), and prognosis in patients treated with immune checkpoint inhibitor (ICI) therapy. ORRs were compared among the non‐users (no PPI and no Abs), single users (PPI or Abs), and double users (PPI and Abs) (A). Occurrences of irAEs were compared among the non‐users, single users, and double users (B). Unadjusted comparison of progression‐free survival (PFS) (C) and OS (D) after ICI therapy among the non‐users, single users, and double users

ORR after ICI therapy was significantly poorer in patients using both PPI and Abs (double users, 12%) than in patients using neither (non‐users, 40%, *p* = 0.004) or patients using either PPI or Abs (single users, 33%, *p* = 0.010) (Figure 3A). The rate of irAEs was not significantly different among the non‐users, single users, and double users (Figure 3B). The PFS after ICI therapy was significantly shorter in the double users (median 3.0 months) than in the non‐users (median 37 months, *p* < 0.001) or single users (median 5.8 months, *p* = 0.035) (Figure 3C). The OS after ICI therapy was significantly shorter in the double users (median 6.5 months) than in the non‐users (median 50 months, *p* < 0.001) or single users (median 15 months, *p* = 0.015) (Figure 3D).

#### 3.3.3 Multivariable Cox regression analyses for PFS and OS

**FIGURE 4:**
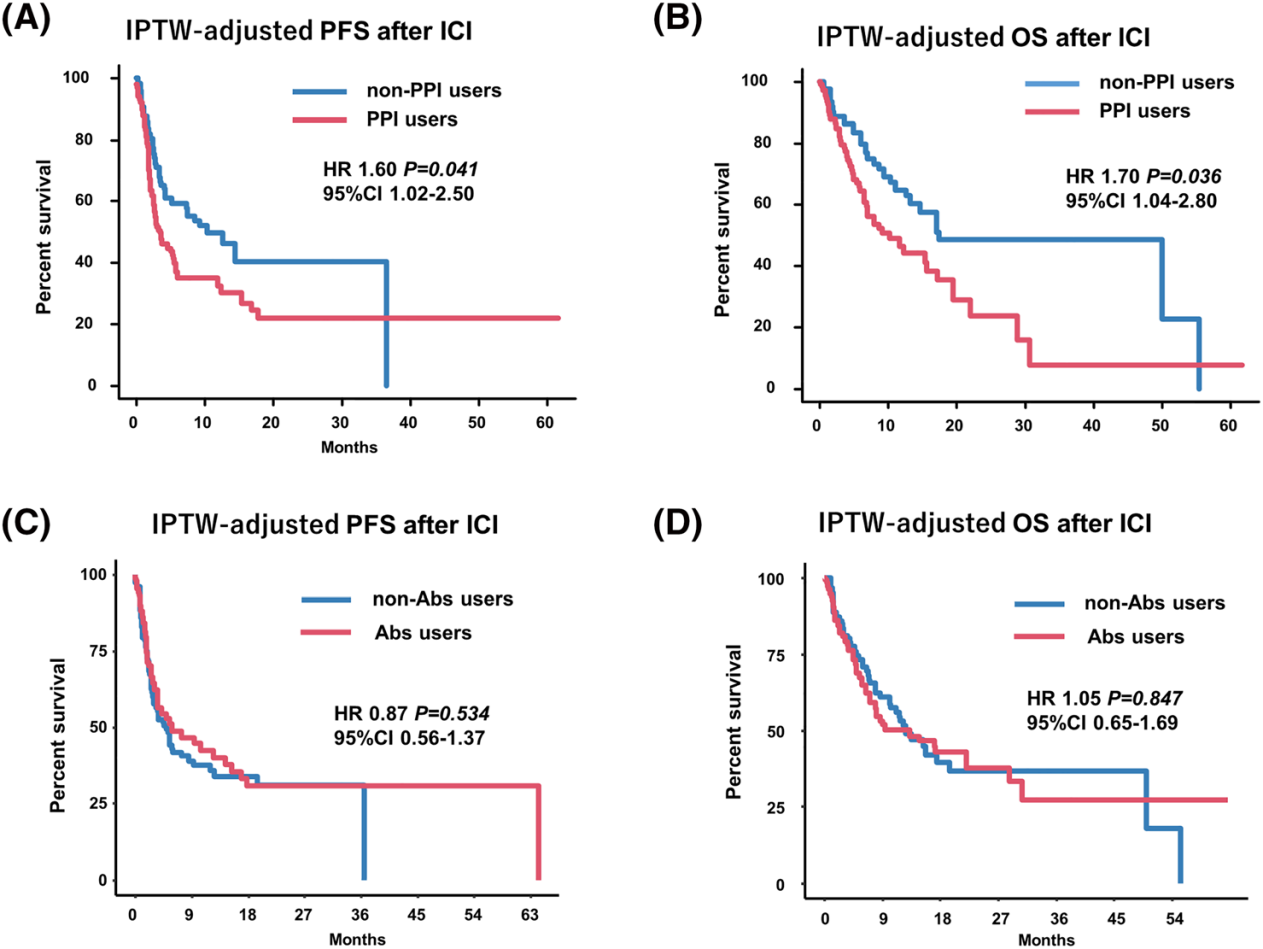
The inverse probability of treatment weighting (IPTW)‐adjusted multivariable Cox regression analysis for progression‐free survival (PFS) and overall survival (OS). Multivariate Cox regression analysis using the IPTW method for PFS (A) and OS (B) after immune checkpoint inhibitor (ICI) therapy between proton pump inhibitor (PPI) users and non‐PPI users. The adjusted variables for the IPTW model were age, sex, ICI therapy treatment line, Eastern Cooperative Oncology Group performance status (ECOG‐PS) at the initiation of ICI therapy, tumor type, Abs use, and exposure to radiotherapy. Multivariate Cox regression analysis using the IPTW method for PFS (C) and OS (D) after ICI therapy between Abs users and non‐Abs users. The adjusted variables for the IPTW model were age, sex, ICI therapy treatment line, ECOG‐PS at the initiation of ICI therapy, tumor type, PPI use, and exposure to radiotherapy

Multivariable Cox regression analyses for PFS and OS was shown in Table 2. Multivariable Cox regression analysis using the IPTW method revealed a significant difference in PFS (HR: 1.60, 95% CI [1.02, 2.50], *p* = 0.041, Figure 4A) and OS (HR: 1.70, 95% CI [1.04, 2.80], *p* = 0.036, Figure 4B) after ICI therapy between the non‐PPI users and PPI users. Multivariate Cox regression analysis using IPTW showed no significant differences in PFS (HR: 0.87, 95% CI [0.56, 1.37], *p* = 0.534, Figure 4D) and OS (HR: 1.05, 95% CI [0.65, 1.69), *p* = 0.847, Figure 4D) after ICI therapy between the non‐Abs users and Abs users. A schematic summary of present study was shown in Figure S1 as a visual abstract.

**TABLE 2:** Multivariable Cox regression analyses for progression‐free survival (PFS) and overall survival (OS)

	Variable	Risk factor	*p* value	HR	95% CI
PFS					
	Age, years	Continuous	0.743	1.00	0.98–1.02
	Gender	Male	0.088	1.54	0.94–2.52
	Type of tumor	UTUC	0.734	0.93	0.59–1.44
	ECOG ‐PS at initiation of ICI therapy	0–4	<0.001	2.12	1.68–2.69
	Exposure to radiotherapy	Yes	0.210	0.66	0.34–1.27
	Number of metastatic sites	0–5	0.001	1.47	1.16–1.85
	ICI therapy treatment line	1–7	0.257	1.19	0.88–1.60
	PPI use	Yes	0.026	1.72	1.07–2.77
	Abs use	Yes	0.105	0.67	0.42–1.09
OS					
	Age, years	Continuous	0.382	0.99	0.97–1.01
	Gender	Male	0.036	1.81	1.04–3.17
	Type of tumor	UTUC	0.696	0.91	0.56–1.47
	ECOG‐PS at initiation of ICI therapy	0–4	<0.001	2.17	1.70–2.77
	Exposure to radiotherapy	Yes	0.250	0.63	0.29–1.38
	Number of metastatic sites	0–5	0.001	1.58	1.21–2.05
	ICI therapy treatment line	1–7	0.506	1.13	0.79–1.60
	PPI use	Yes	0.039	1.78	1.03–3.07
	Abs use	Yes	0.949	1.02	0.63–1.65

Abs, antibiotics; CI, confidence interval; ECOG‐PS, Eastern Cooperative Oncology Group performance status; HR, hazard ratio; ICI, immune checkpoint inhibitors; PPI, proton pump inhibitors; UTUC, upper tract urothelial carcinoma.

## 4 DISCUSSION

This study investigated the prognostic impact of the concomitant use of PPI and/or Abs on oncological outcomes in patients with advanced UC treated with ICI. ORR was significantly lower in patients who used PPIs, whereas Abs use did not affect ORR. The background‐adjusted PFS and OS after ICI therapy were significantly shorter in patients who used PPI, which was consistent with the results of a post‐hoc analysis of clinical trials.^13^ In contrast to the post‐hoc clinical trial analysis, we did not observe any effects of Abs on PFS and OS after ICI therapy. The reason for this discrepancy is unclear; however, the limited effects of Abs on prognosis might be related to differences in the duration of PPI and Abs use. In our practice, we administrated a single‐ or 1‐day dose of Abs for invasive procedures, such as biopsy or transurethral resection. Conversely, we administrated PPI continuously before and after ICI therapy if a patient had gastroesophageal reflux or peptic ulcers. However, we could not address the impact of the duration of PPI and Abs treatment because of a lack of data. Also, the cohort is underpowered to discern the differences between the peri‐procedural and other prophylaxis. Although both PPI and Abs are potentially associated with gut microbiota dysbiosis, not enough information is available for the effect of both PPI and Abs on gut microbiota dysbiosis. Further study is necessary to address this issue.

Reports concerning the effects of dysbiosis‐inducing drugs are conflicting. Recent studies have suggested that exposure to corticosteroids, antibiotics, and PPI leads to progressively poorer outcomes after ICI therapy.^22^, ^23^ Conversely, a meta‐analysis that included 1167 patients with cancer examined the effect of PPI on the survival of patients with cancer treated with ICIs; this study showed that the concomitant use of PPI did not significantly affect OS (HR: 0.996; 95% CI [0.486, 1.447]) or PFS (HR: 0.858; 95% CI [0.388, 1.328]).^24^ These contentious outcomes stem mainly from the indirect analysis of the effects of dysbiosis‐inducing drugs on oncological outcomes. Some types of gut microbiota may play a key role in tumor responses.^25^, ^26^ In addition, PPI use can induce a significantly lower abundance of gut commensals and microbial diversity within 4 weeks, with a significant change in the abundance of *Lactobacillus* and *Streptococcus* species.^12^, ^27^ However, the immunological functions of these bacteria remain unclear and the presence or absence of specific types of bacteria among the complex gut microbiota could not explain the effects of PPI on ICI therapy response.

The accumulation of dysbiosis‐inducing drugs was strongly associated with poor PFS and OS after ICI therapy. We found that patients who used both PPI and Abs exhibited significantly poor ORR, PFS, and OS after ICI therapy. A similar observation was reported in a recent study that included 1229 patients with non–small‐cell lung cancer (70%), melanoma (14.7%), renal cell carcinoma (9.2%), and others (6%).^22^ The authors of the study developed a drug‐based prognostic score that included six baseline medications (corticosteroids: 2 points, antibiotics: 1 point, and PPI: 1 point); patients with a high score had significantly poorer prognoses than those with a low score.^22^ Therefore, we need to be careful for the accumulation of dysbiosis‐inducing drugs in patients with ICI therapy.

The use of corticosteroids is associated with irAEs in patients treated with ICI therapy. A previous study suggested that the use of corticosteroids reduces the efficacy of ICI therapy via reduction of CD8 + T cell proliferation.^28^ In addition, PPI‐driven gastric hypochlorhydria was associated with the promotion of T cell tolerance and the acidic microenvironment of tumor cells, which facilitates the proliferation, progression, and metastasis of tumors.^29^ Therefore, the combination of dysbiosis‐inducing drugs and immune suppression agents for the drug‐based prognostic score is reasonable. However, the precise mechanisms of the effect of these agents on ICI therapy remain unclear and require further study.

There is a lack of studies investigating the effects of the concomitant use of PPI or Abs and ICIs on irAEs. As many reports suggested a positive association between the efficacy and immune‐related AEs,^6^, ^30^ we speculate that the concomitant use of PPI or Abs may reduce the incidence of irAEs in conjunction with attenuated therapeutic effects. However, we observed no significant association of the concomitant use of PPI or Abs with the incidence of irAEs. We found a similar proportion of irAEs in PPI users (37%), Abs users (35%), and double users (34%) (Figure 3B). We could not explain this discrepancy, although there may be an interaction between efficacy, PPI/Abs use, and irAEs. Further research is needed to understand the interaction between efficacy and irAEs in patients undergoing ICI therapy.

Several limitations in this study should be acknowledged. First, because of the retrospective study design, we could not control for selection bias and other unmeasurable confounders. Second, the statistical analysis might be underpowered because of the small sample size. Analyses under a single population are a problem for generalization. Third, lack of data on gut microbiota and corticosteroid use are robust limitations of this study. Forth, we could not address the duration of PPI/Abs treatment and the type of Abs. Nonetheless, this study demonstrates the effect of concomitant PPI and/or Abs use on prognosis in patients with advanced UC treated with ICI therapy. Thus, unnecessary PPI or Abs should be avoided for patients with advanced UC who require ICI therapy. Further prospective studies are required to determine the role of concomitant use of PPI and Abs in the reduced antitumor immune responses via gut dysbiosis.

## 5 CONCLUSIONS

The concomitant use of PPI may adversely affect oncological outcomes in patients with locally advanced or metastatic UC treated with ICI therapy.

## FUNDING INFORMATION

This study was supported by Japan Society for the Promotion of Science (JSPS) KAKENHI (grants 19H05556 [C. O.], 20K09517 [S. H.], 19K18603 [N. F.], and 18K16718 [D. N.]).

## CONFLICT OF INTEREST

The authors have no conflict of interest.

## AUTHORS' CONTRIBUTION

Yoshiharu Okuyama is responsible for data collection. Shingo Hatakeyama is responsible for project development, manuscript editing, data analysis, and data collection.

Kazuyuki Numakura, Takuma Narita, Toshikazu Tanaka, Yuki Miura,

Daichi Sasaki, Daisuke Noro, Noriko Tokui, Teppei Okamoto, Hayato Yamamoto, Shintaro Narita, Takahiro Yoneyama, Yasuhiro Hashimoto are involved in data collection. Tomonori Habuchi and Chikara Ohyama are involved in project development and critical review.

## Supporting information


**Figure S1.** Visual abstract
A schematic summary of present study was shown.
