# Supplementary figures and images for: Prognostic impact of proton pump inhibitors for immunotherapy in advanced urothelial carcinoma
Source: BJUI Compass. 2021 Oct 8;3(2):154–61. doi: 10.1002/bco2.118 (PMC8988833; doi:10.1002/bco2.118)

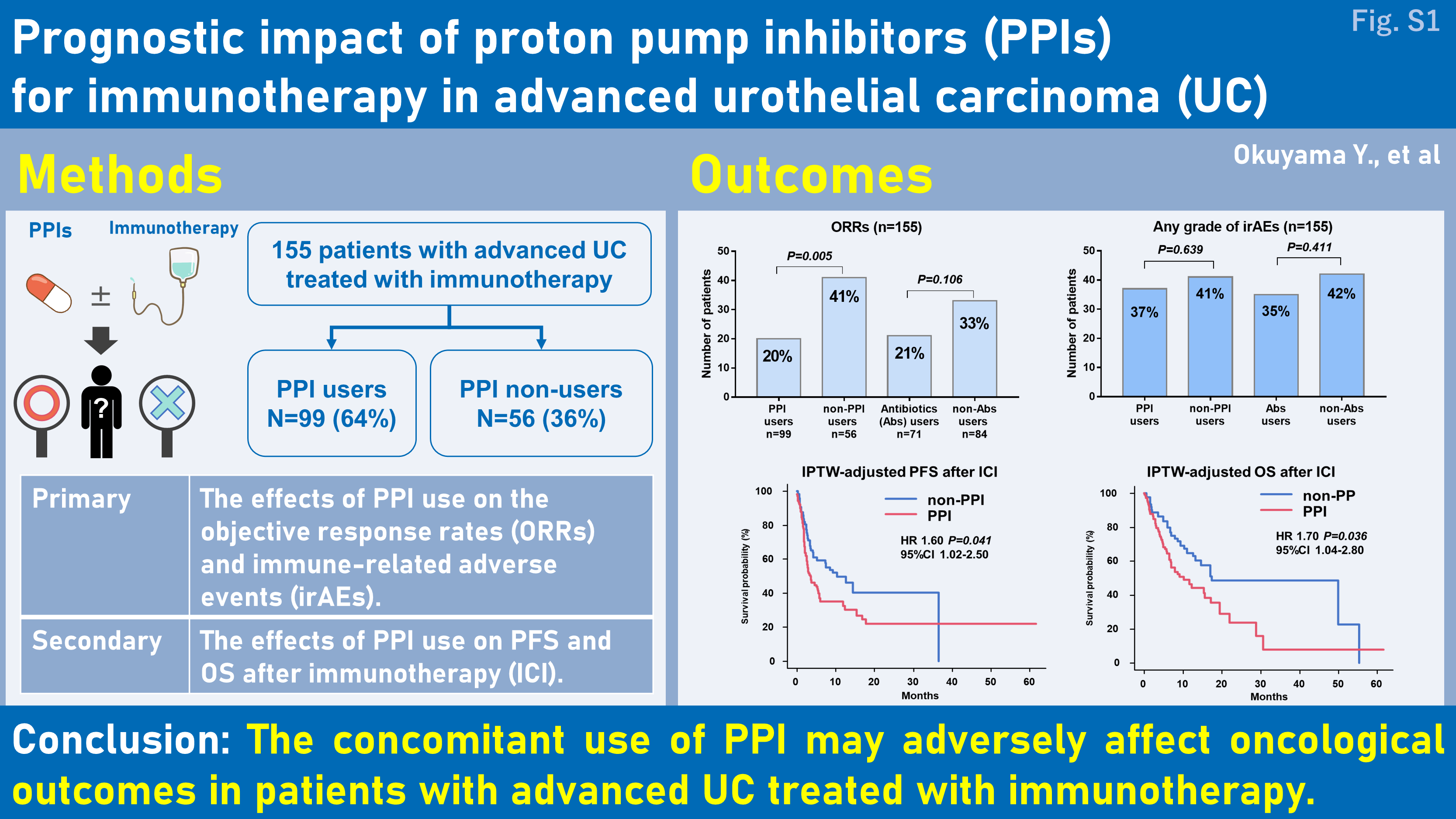

Supplement: Supplementary file 1 — Figure S1. Visual abstract A schematic summary of present study was shown. [file BCO2-3-154-s001.tif]
